# The effect of changing micro-scale physical environmental factors on an environment’s invitingness for transportation cycling in adults: an exploratory study using manipulated photographs

**DOI:** 10.1186/s12966-014-0088-x

**Published:** 2014-08-19

**Authors:** Lieze Mertens, Veerle Van Holle, Ilse De Bourdeaudhuij, Benedicte Deforche, Jo Salmon, Jack Nasar, Nico Van de Weghe, Delfien Van Dyck, Jelle Van Cauwenberg

**Affiliations:** Department of Movement and Sport Sciences, Faculty of Medicine and Health Sciences, Ghent University, Watersportlaan 2, B-9000 Ghent, Belgium; Department of Human Biometry and Biomechanics, Faculty of Physical Education and Physical Therapy, Vrije Universiteit Brussel, Pleinlaan 2, B-1050 Brussels, Belgium; Research Foundation Flanders (FWO), Egmontstraat 5, 1000 Brussels, Belgium; Centre for Physical Activity and Nutrition Research, School of Exercise and Nutrition Science, Deakin University, Burwood Highway 221, Burwood, VIC 3125 Australia; Ohio State University, City and Regional Planning, 292 Knowlton Hall, West Woodruff Avenue 275, Columbus, OH 43210 USA; Department of Geography, Faculty of Sciences, Ghent University, Krijgslaan 281, S8, B-9000 Ghent, Belgium

**Keywords:** Built environment, Biking, Adulthood, Experiment, Pictures, Transport, Physical activity

## Abstract

Previous studies have shown convincing evidence for positive relationships between transportation cycling in adults and macro-scale physical environmental factors. In contrast, relationships are less consistent for more changeable, micro-scale environmental factors. The majority of existing studies used observational study designs, which cannot determine causality. The present mixed-methods study used manipulated photographs to determine causal relationships between micro-scale environmental factors and the environment’s invitingness for transportation cycling. Further, interactions among environmental factors and moderating effects of gender, age and educational level were investigated. For this study, panoramic photograph of a street was manipulated on eight environmental factors: traffic, speed bump, general upkeep, evenness of the cycle path, vegetation, separation of motorized traffic, separation with sidewalk and cycle path width. Sixty-six middle-aged adults participated in the study and sorted the manipulated panoramic photographs from least to most inviting to cycle for transportation. Participants also provided qualitative data on how they sorted the streets. Multilevel cross-classified modelling was used to analyse the relationships between the environmental manipulations and the invitingness-scores. The qualitative data were deductively categorized according to the environmental factors. All environmental factors, except for separation with sidewalk, proved to have a significant main effect on the invitingness-score for transportation cycling. Cycle path evenness appeared to have the strongest effect on the invitingness. This effect was even stronger in an environment with good compared to poorly overall upkeep. Another significant interaction effect showed that the invitingness decreased when both separations along the cycle path were present compared to only a separation with traffic. No moderating effects of the demographic factors on these relationships were found. Qualitative data confirmed the observed quantitative relationships and added depth and understanding. Current study shows that the use of manipulated photographs can be an effective way to examine environment-physical activity relationships. Our findings indicate that evenness of the cycle path may be a crucial environmental factor when aiming to increase a street’s invitingness for transportation cycling among middle-aged adults. The findings of our exploratory study could be used to develop an environmental intervention to determine if our findings are applicable to real changes in cycling behavior.

## Background

Although the benefits of physical activity (PA) are well-known in many countries around the world, approximately 31% of adults (15 years and over) do not reach the public health guideline of 150 min/week of moderate-to-vigorous PA [1]. Interventions that focus on the incorporation of PA into daily routines are required. One possible solution is to incorporate the habitual use of active transport into daily routines. Cycling for transportation has many health benefits, and is also an important behavior from economic, social, environmental and traffic management perspectives [2-11]. Moreover, cycling for transportation has the potential to increase PA levels in European adults. Despite the many benefits, more than 30% of all trips made by car in Europe cover distances of less than 3 km and 50% are shorter than 5 km [12]. It is therefore important to identify reasons why people do and do not cycle for transport. Socio-ecological models state that the physical environment, together with social and individual attributes, provide a useful framework for explaining active transportation [13].

In previous cross-sectional studies, positive relationships between environmental factors such as walkability, access to shops/services/work, and urbanization and transportation cycling in European [14] and non-European [15] adults have been reported. These macro-scale environmental factors may be difficult to change in existing neighborhoods. In contrast, relationships are less consistent for more changeable, micro-scale environmental factors, such as vegetation, upkeep, evenness of the cycle path or traffic-related safety [14,16-18]. A possible explanation for these inconsistencies may be that environmental perceptions are generally assessed using questionnaires. Although usually valid and reliable tools are used, there are disadvantages of using questionnaires to assess features of the physical environment. Firstly, participants need to recall features of the physical environment and often, important environmental attributes are overlooked, neglected or forgotten due to recall bias [19]. Secondly, there is no standard definition of “the neighborhood”, which can cause a mismatch between the target environment of the researcher and that of the participant. To accommodate these disadvantages, the use of photographs may serve as an appropriate alternative for investigating the physical environment. Furthermore, these inconsistencies may be addressed by collecting qualitative data, which enables a more in-depth understanding of what people are thinking about when they rate their neighborhood environment. Very little built environment research has utilized a mix of qualitative and quantitative methodologies [13,20].

A major limitation of previous research of the physical environment is that most studies have used observational study designs, which are not suitable for determining causality [21-23]. Because it is often not feasible to change the real environment within a research context, an experimental design using photographs and manipulating environmental factors depicted in these photographs can offer a suitable solution to identify causal relationships with the invitingness for transportation cycling. Manipulating photographs instead of real-life environments allows changing of factors or combinations of environmental factors such as evenness of the cycle path, vegetation, upkeep and traffic level while other factors are standardized. In contrast, questionnaires only have the possibility of asking one item at a time, while the real environment consists of a combination of several environmental factors simultaneously. Therefore, it is also important to investigate the moderating effects of environmental factors on the relationship between another environmental factor and the invitingness for transportation cycling. For example, the presence of a separation between cycle path and motorized traffic might only enhance the invitingness for transportation cycling if much traffic is present compared to no traffic. Conversely, the presence of vegetation might be stronger in a well-maintained compared to a poorly maintained environment. Findings obtained from research using photographs could inform environmental interventions in real life settings about which micro-scale environmental factors to modify.

A further aspect that has been infrequently studied is the moderating role of demographic factors on the relationships between micro-scale environmental factors and the likelihood of cycling for transportation. Previous research has shown that cycling for transport differs between men and women, age and socioeconomic status [24-26]. These sub-group differences in cycling for transport may be explained in part by differences in perceptions and engagement with the built environment [27-29]. Experimental research using mixed methods approaches may be useful for examining whether these demographic factors moderate associations between the built environment and cycling for transport.

The first aim of the current study was to examine the effect of manipulating photographs of micro-scale physical environmental features on adults’ perceptions of the environmental invitingness for transportation cycling. Secondly, interactions among environmental factors on the invitingness were investigated to identify if certain micro-scale environmental factors moderate the relationships between other environmental factors and the invitingness for transportation cycling. Finally, moderating effects of gender, age and educational level were investigated. Both quantitative and qualitative information was collected to answer these questions.

## Methods

By purposeful convenience sampling, 66 Flemish middle-aged adults (45–64 years), stratified by gender, were recruited. Only middle-aged adults living in an urban (>600 inhabitants/km^2^) or semi-urban (300–600 inhabitants/km^2^) municipality [30] in the region of Flanders or the Brussels Capital Region were eligible. These recruitment areas were chosen because average trip distance corresponds with a 10-minutes cycle trip and participants were required to imagine such trips in the measurement protocol (see below). Furthermore, the participants had to be physically able to cycle for 30 minutes and only one person per household could participate.

### Protocol and measures

Participants were visited at home by a researcher between March and April 2013 and completed a two-step research protocol. Before starting the measurement, informed consent was obtained from the participants. The home visit consisted of a structured interview and a sorting task with panoramic photographs which lasted approximately one hour. This study protocol was approved by The Ethics Committee of the University Hospital. The detailed protocol is described below.

### Photograph development

Prior to data collection, two sets (set A and set B) of 32 panoramic color photographs were developed with Adobe Photoshop^©^ software. Previous research has established the validity of responses to color photos in comparison to on-site responses [31-33]. The use of programs such as Adobe Photoshop^©^ to create controlled and realistic manipulations of the physical environment has been proposed by Nasar [32]. To enhance standardization, each photograph depicted the same street taken from an adult cyclist’s eye-level viewpoint under dry weather conditions and without people visible in the environment. Each panoramic photograph differed from the others in at least one environmental manipulation. Five possible manipulated environmental factors were present in each photograph and were depicted in two categories: presence (score 1) or absence (score 0) of the positive environmental characteristic. The presence or absence of manipulations of five environmental factors led to a total of 32 (2^5^ = 32) images per set. As adding an additional environmental factor would double the number of pictures and would overload the participants, two separate sets of photographs were made (set A and set B), so that a total of eight different environmental factors could be examined, including two factors that were used in both sorting tasks. Each photograph was 10.63 inches (27 cm) wide and 2.36 inches (6 cm) high.

**Figure 1:**
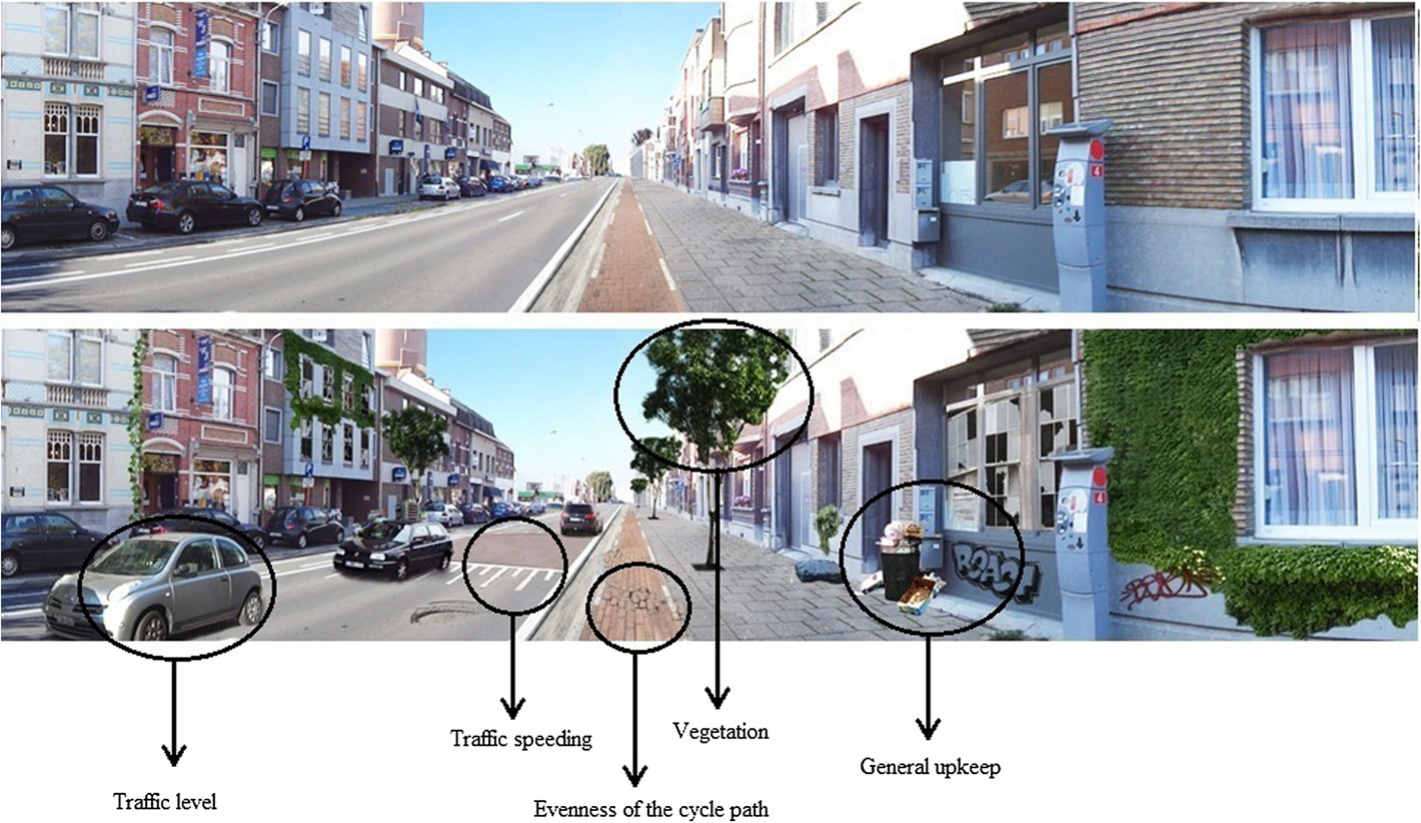
**Examples of manipulated photographs from set A with 5 environmental factors manipulated.**

Based on previous research with non-manipulated panoramic photographs [34] and existing literature on environment-transportation cycling relationships [14,35], the key factors for adults’ cycling for transportation could be determined. The following five factors were selected to be manipulated in set A: ‘traffic level’ , ‘traffic calming’ , ‘the evenness of the cycle path’ , ‘general upkeep’ and ‘vegetation’ (Figure 1). ‘Traffic level’ was manipulated by the presence or absence of driving cars on the road. ‘Traffic calming’ was manipulated by the presence or absence of a speed bump. The ‘evenness of the cycling path’ was manipulated by depicting a cycle path in good condition or in poor condition. ‘General upkeep’ represented the overall maintenance degree of the depicted environment and was manipulated by putting graffiti on the wall, depicting broken windows, garbage on the street and a hole in the road surface. Finally, the presence or absence of trees along the road and greenery on houses were the manipulations done for ‘vegetation’.

**Figure 2:**
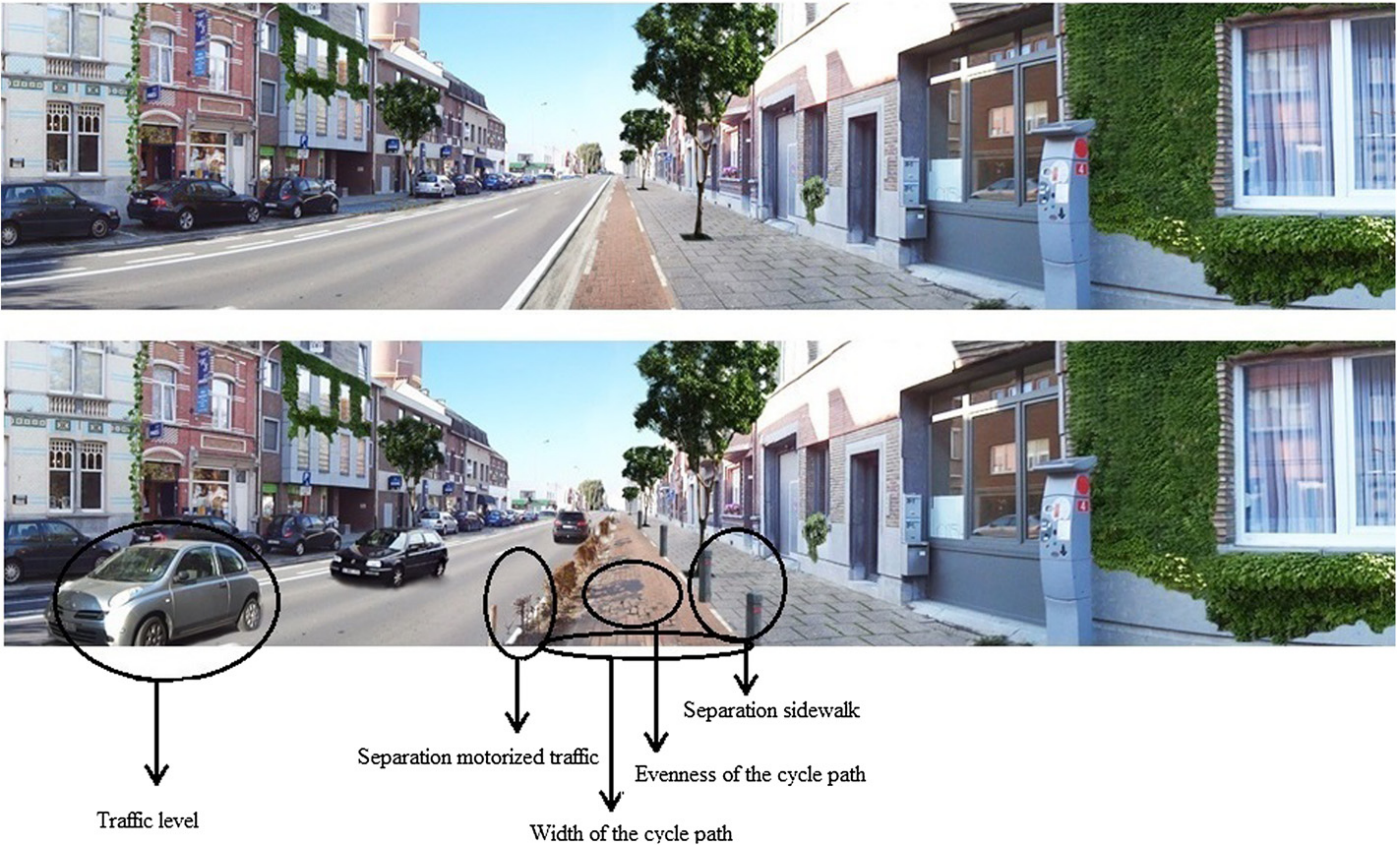
**Examples of manipulated photographs from set B with 5 environmental factors manipulated.**

Besides the first set of photographs including the key factors determined from previous research and literature, set B was developed to determine the effect of traffic safety elements on the invitingness for transportation cycling, which has been already reported in previous studies as important to obtain higher levels of cycling [14,35,36]. Therefore, two environmental factors were manipulated in set A as well as in set B, namely ‘traffic level’ and ‘evenness of the cycle path’. The other three environmental factors manipulated in set B were: ‘separation between cycle path and motorized traffic’ , ‘separation between cycle path and sidewalk’ and ‘width of the cycle path’ (Figure 2). The ‘separation between cycle path and motorized traffic’ was manipulated by whether or not a hedge was present between these two. In contrast, the manipulation of the ‘separation between cycle path and sidewalk’ was done by the presence or absence of bollards between the two. Finally, the width of the cycle path was manipulated by depicting a narrow or wide cycle path.

### Interview

The home visit started with a short face-to-face interview, assessing sociodemographic information (gender, age, country of birth, highest degree of education, occupational status, marital civil status, number of vehicles in the household) and PA level. PA level was measured with the International Physical Activity Questionnaire (IPAQ, long, last 7 days interview version) [37]. Self-reported PA assessed by IPAQ showed good reliability (Spearman’s correlation coefficients clustered around 0.80) and acceptable criterion validity (median ρ = 0.30) for middle-aged adults in a 12-country study [38]. Only the domains of PA that are potentially affected by the neighborhood environment (i.e., active transportation and recreational PA) were surveyed.

### Sorting task

During the home visit, the participants were asked to do two similar sorting tasks (one for each set of 32 colored photographs). Before starting the sorting task, the researcher randomly scattered all 32 photographs on a table and read the following standardized instructions out loud: *‘Imagine yourself cycling to a friend’s home located at 10 minutes cycling from your home during daytime with perfect weather. First, it is intended that you pick the worst and the best street(s) to cycle along to the house of your friend. There is no good or wrong solution, we are only interested in what matters to you the most while cycling to your friend’.* When the participant had chosen the most and least inviting environments, the researcher spread 11 cards depicting scores ranging from zero to ten on the table. The following standardized instructions were given to sort the environments on their invitingness for transportation cycling on an 11-point Likert scale ranging from 0 (not inviting at all), through 5 (neutral), to 10 (very inviting): *“The photograph(s) that you indicated as least inviting were placed under score 0 and the most inviting photograph(s) under score 10. Now you have to sort the remaining pictures from lowest to highest invitingness by assigning them a score from zero to ten. You can place several pictures under the same score and you can switch them every moment. You can still move the pictures that have already received a score of 0 or 10 or add other photographs to these scores”.* To identify the reasons for sorting the photographs in that way, qualitative information was collected. The next part of the study was recorded by a voice-recorder and the participants were asked to describe the reasons why they had sorted the pictures in that way. If necessary, the researcher prompted for further explanation. For the other set of 32 photographs, the same protocol was followed. To prevent order effects, the protocol alternately started with set A or B between participants.

### Analyses

#### Quantitative analyses

Descriptive statistics were performed using SPSS 20.0 software. Multilevel cross-classified linear regression models in MLwiN 2.28 [39] were used to analyze the quantitative data to account for clustering of the invitingness-scores within participants and streets (participants and streets were treated as cross-classified) [40]. Markov Chain Monte Carlo (MCMC) procedures were used for model parameter estimation [41].

A final model was constructed in five phases. In a first step the main effects of age, gender and education on the assigned invitingness scores were analyzed in three separate models. Secondly, a basic model was developed that included the five environmental factors and the individual factors that were significantly related to the invitingness scores in step 1. Thirdly, interaction effects between environmental factors and individual factors and between environmental factors mutually were added to the basic model. In the last step, all significant main and interaction effects obtained from previous phases were combined into one model. The final model was constructed by allowing random slopes and by deleting non-significant effects that did not improve the model fit. Models were compared using the Deviance Information Criterion (DIC) [42]. This procedure was performed separately for photograph sets A and B. Level of significance was defined at α = 0.05.

### Qualitative analyses

The first step in the analysis of the qualitative data involved reading the transcripts in detail. Nvivo 9 Software (QRS International) was used to categorize qualitative data into five categories corresponding to the five manipulated environmental factors [43]. This categorization was based on the framework approach as presented by Pope and colleagues [44]. Finally, the data were summarized by environmental factor. This procedure was accomplished separately for photograph sets A and B. Because the environmental factors ‘traffic level’ and ‘evenness of the cycle path’ were manipulated in both sorting tasks, the qualitative data collected from sorting task A and B for these factors were analyzed together. Quotes from participants were used to clarify the findings.

## Results

### Descriptive statistics

In total, 66 adults ranged in age from 45 to 65 years participated in the study. Just over half of the sample were women and more than half attended university. Just one in four participants met PA recommendations and only one in five reported cycling for transport in the last seven days. Other descriptive characteristics of the sample are shown in Table 1.

**Table 1:** **Descriptive characteristics of the participants (n = 66)**

**Age (M ± SD)**	53.6 ± 5.0
**Women (%)**	53.0
**Born in Belgium (%)**	95.5
**Marital status (%)**	
-Married	77.3
-Widowed	4.5
-Single	12.1
-Cohabiting	6.1
**Education (%)**	
-Primary	6.1
-Lower secondary	39.4
-Higher secondary	54.5
-Tertiary	31.7
**Occupational status (%)**	
-Household	9.1
-Blue collar	19.7
-White collar	71.2
**Physical activity**	
-Moderate-to-vigorous PA min/wk (M ± SD)	114.1 ± 167.6
-Meeting PA recommendations (%)	25.8
**Current cycling for transportation level**	
-Cycling for transportation min/wk (M ± SD)	32.8 ± 76.1
-No cycling for transportation (%)	80.3

M = mean; SD = standard deviation.

### Quantitative analyses

**Figure 3:**
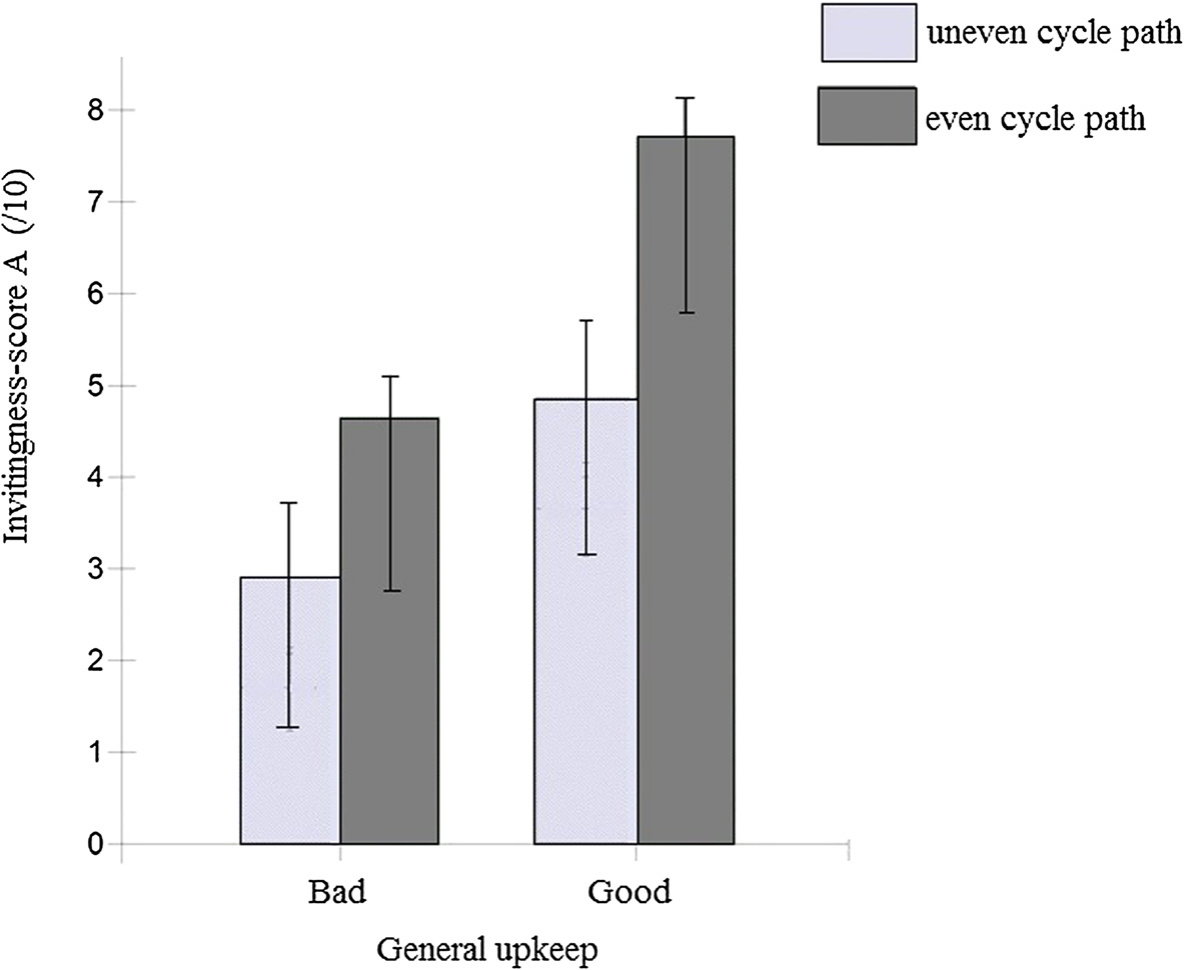
**Interaction effect of ‘cycle path evenness’ and ‘general upkeep’ on the invitingness-score (sorting task A).**

For sorting task A, the final model showed that all five environmental factors were significantly related to the invitingness for transportation cycling (Table 2). No traffic, the presence of a speed bump, an even cycle path, a well-maintained environment and the presence of vegetation increased perceived invitingness for transportation cycling. The largest change of the invitingness-score of transportation cycling was found between an environment with an uneven compared to an even cycle path, with an increase of 2.52 ± 0.35 points on a 11-point Likert-scale (range 0–10). Furthermore, one significant interaction effect was found, namely between ‘evenness of the cycle path’ and ‘general upkeep’ (p < 0.001). The positive effect of evenness is greater if the environment is well-maintained, compared to when it is poorly maintained (Figure 3). No moderating effects of gender, age and degree of education were found.

**Table 2:** **Main and interaction effects of the environmental and demographic factors**

***Sorting task A***	**β (S.E.)**	***Sorting task B***	**β (S.E.)**
Intercept	1.12 (0.25)	Intercept	0.60 (0.51)
**Main effects** ^**1**^		**Main effects** ^**1**^	
Traffic level	1.43 (0.21)***	Traffic level	1.26 (0.20)***
Traffic calming	0.35 (0.12)**	Separation MT	1.92 (0.26)***
Evenness of the cycle path	2.52 (0.35)***	Separation sidewalk	−0.45 (0.24)
General upkeep	1.97 (0.24)***	Evenness of the cycle path	3.29 (0.25)***
Vegetation	0.81 (0.16)***	Width of the cycle path	0.78 (0.11)***
**Interaction effects**		**Interaction effects**	
Evenness*general upkeep	1.07 (0.17)***	Separation MT* separation sidewalk	−0.42 (0.13)***

SE = standard error; MT = motorized traffic.

*p < 0.05, **p < 0.01, ***p < 0.001.

^1^The reference categories for the environmental factors were the negative environmental characteristic of the factors (i.e. high traffic level, no speed bump, uneven cycle path, poorly upkeep, no vegetation, no separation MT, no separation sidewalk, narrow cycle path).

Note: The outcome variable of both sorting tasks was the environment’s invitingness-score for transportation cycling on a Likert scale ranging from 0–10. The final model of sorting task A was adjusted for gender and education, since these were found to be related to the invitingness-scores. Similarly, the final model for sorting task B was adjusted for education.

**Figure 4:**
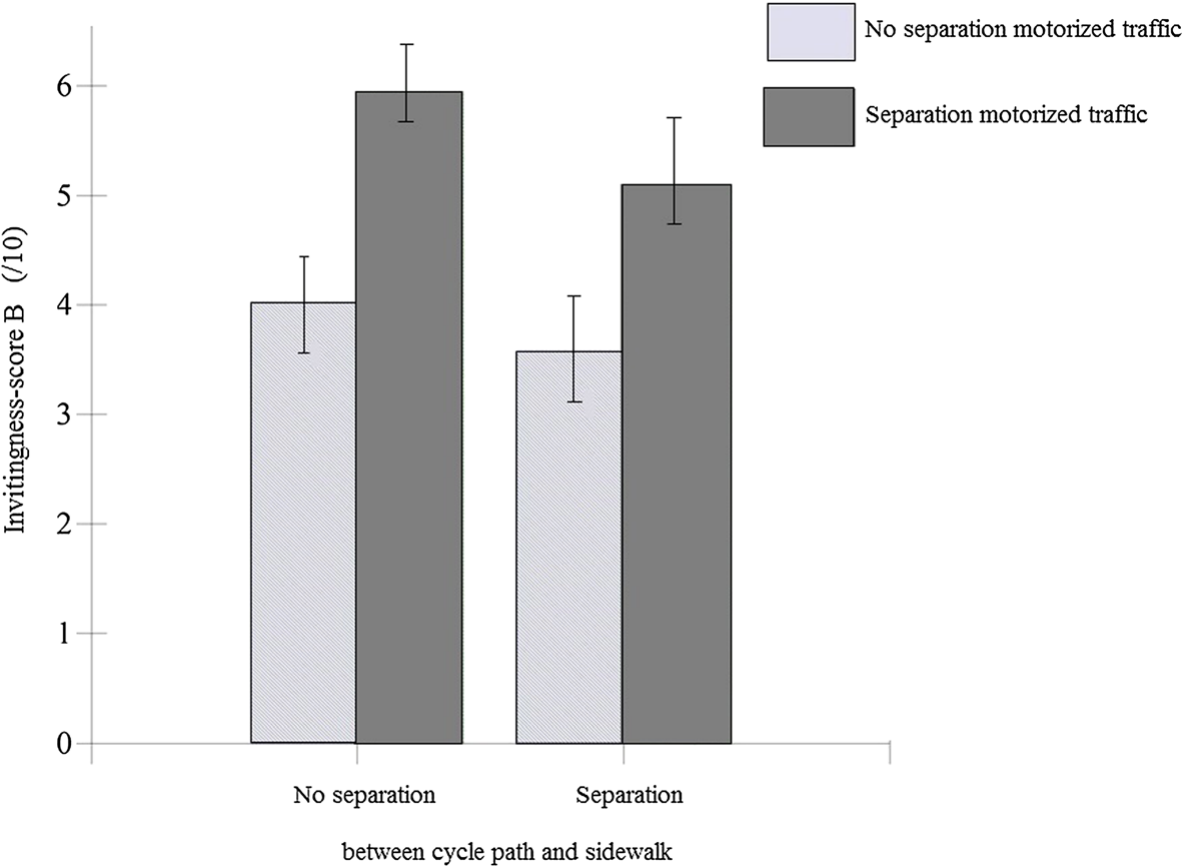
**Interaction effect of ‘separation motorized traffic’ and ‘separation sidewalk’ on the invitingness-score (sorting task B).**

For sorting task B, four of the five environmental factors showed a significant positive main effect on invitingness (Table 2). No traffic, the presence of a separation with motorized traffic, an even cycle path, and a wide cycle path significantly increased the invitingness for transportation cycling. An even cycle path increased the invitingness-score the most with an increase of 3.29 ± 0.25 points on a 11-point Likert-scale (range 0–10). ‘Separation between cycle path and sidewalk’ had no significant main effect (p = 0.062). A significant interaction effect was found between ‘separation between cycle path and motorized traffic’ and ‘separation between cycle path and sidewalk’ (p = 0.001) (Figure 4). A separation between cycle path and sidewalk has a negative effect on the invitingness-score, when there is already a separation between cycle path and motorized traffic present and furthermore, had no effect when a separation between cycle path and motorized traffic was absent. No moderating effects of gender, age and degree of education were found.

### Qualitative analyses

The qualitative information for each environmental factor is described below.

### Traffic level (sorting task A and B)

Participants preferred streets without traffic compared to streets with traffic; however, it was not reported as the most important factor and was often regarded as a temporary situation. The next quote illustrates this clearly: *“First of all, the most important factor is the condition of the cycle path. The traffic that is present, is taken into account, but not so much because it is actually a snapshot, the picture may be completely different five minutes later because those cars can be gone by then, on the other hand it can also be a lot busier by then.” (man, 48 years)*

Other participants considered the presence of traffic from a more realistic perspective: *“The best picture is traffic free, no cars are driving there at the moment, so that gives a safe impression. However, it is not realistic that all streets are free from traffic.” (man, 57 years)*

### Evenness of the cycle path (sorting task A and B)

The participants had a clear and consistent opinion concerning the ‘evenness of the cycle path’: the condition of the cycle path was considered a priority. Fear of falling and other safety components related to the condition of the cycle path, appear to have a great impact on the invitingness: *“The least inviting streets depend on the condition of the cycle path. Cycling becomes more difficult because of the age, resulting in a higher importance of stability and balance. A good cycle path is therefore the most important factor.” (Woman, 54 years)*

*“The most important issue is the pavement and the condition of the cycle path. Safety comes first.” (man, 48 years)*

Also in combination with other environmental factors, evenness of the cycle path was regarded as the most important attribute. This is illustrated by the following quotes: *“I still prefer a good cycle path with a lot of traffic on the road, compared to a bad cycle path without any cars on the road.” (Man, 60 years)*

*“The least inviting pictures are the pictures with a poor cycle path condition. Then I do not distinguish whether there is a speed bump or not because I argue that if the cycle path is not even, cyclists may fall. For me, that was the most important criterion.” (woman, 54 years)*

### Traffic calming (sorting task A)

The negative relationship between the presence of a speed bump and the speed of cars was mentioned by a few participants but was not considered as very important because the presence of a speed bump indirectly shows that many cars drive in the street. This is illustrated by the following quotes: *“A speed bump in the street, is less important for cyclists, because cars still drive there anyway. The fact that there is a speed bump is a mitigating factor but is less important.” (Man, 53 years)*

Furthermore, some participants also mentioned a disadvantage of a speed bump, as cited in the following quote: *“The speed bump, either it bothers a little because of the annoying noise when cars driving over, or it is good when it slows down the speed of cars.” (Woman, 64 years)*

### General upkeep (sorting task A)

Many participants considered a poorly maintained environment as uninviting to cycle because it is not attractive or they feel unsafe. The following quotes illustrate this: *“So the pictures that I did not find attractive are the streets that are very sloppy. The establishments are also untidy and I feel unsafe. I am most attracted to the pictures where everything is clean. Both the cycle path, the street and the establishments are well-maintained. These are actually the criteria that are important for me.” (woman, 53 years)*

The garbage was often mentioned as a possible obstacle while cycling, or for pedestrians who would move to the cycle path and hinder cyclists while they avoid the garbage. The quote below illustrates the attention that participants paid to the hole in the road: “*The criteria used to choose the least inviting street includes the poor condition of the road (hole in the road surface), because of the risk that cars will swing out to the cycle path to avoid the hole. That was a very important thing.” (Woman, 50 years)*

### Vegetation (sorting task A)

‘Vegetation’ was not considered to be a priority for the participants, but rather an additional component. The next quote illustrates this: *“What I really do not like is the broken cycle path. The green on the side, the bushes and the trees, I find enjoyable but that is not really a priority. Safety is more important.” (Woman, 58 years)*

The presence of trees was not always reported as increasing invitingness to cycle. Participants often saw it as an obstacle while cycling, or as an obstacle for pedestrians who would move to the cycle path, as mentioned in the following quote: *“This is the least inviting picture because the cycle path is uneven, there is quite a lot of traffic on the road and there are trees on the sidewalk. I think pedestrians can switch to the cycle path and disturb cyclists.” (Woman, 51 years)*

### Separation between cycle path and motorized traffic (sorting task B)

Regarding the presence of a ‘separation between cycle path and motorized traffic’ , many participants agreed that it provides an important protection for cyclists and that it increases rider safety. Separation from motorized traffic was generally preferred compared to no traffic protection: *“This picture is more inviting to cycle because effort is made to draw a border between cyclists and cars.” (woman, 50 years)*

However, some adults did not like the presence of a separation on both sides of the cycle path because this gives a frightening feeling, especially in combination with a narrow bike path. This is illustrated by the following quote: “*What appears to be negative for me is having a separation on both sides of the cycle path. This is just a little too generous and moreover gives me a feeling of tightness. The most frightening separation is the separation to the sidewalk, the positive one is the separation to the street because it protects you from cars.” (woman, 49 years)*

### Separation between cycle path and sidewalk (sorting task B)

Most participants did not like the separation between cycle path and sidewalk because of the bollards that were used to distinguish footpath and cycle path. They were seen as uninviting, as an obstacle giving limited evasive options or giving the feeling that you were pushed to the street. This is described in the next quote: *“For the least inviting environments, I have taken the pictures with the bollards. I really do not like the bollards because I would automatically go driving on the road instead of the cycle path, just to avoid the bollards.” (Woman, 53 years)*

### Width of the cycle path (sorting task B)

People preferred a wide bike path compared to a narrow one, but this was not considered as a priority. This is mentioned in the following quote: “*In the first place, I have watched the condition of the cycle path. Secondly, I made a distinction in whether or not there was a separation between cycle path and traffic. Afterwards, I looked whether or not the bike path is wide.” (Man, 53 years)*

## Discussion

This study examined the effect of manipulating micro-scale physical environmental factors on an environment’s perceived invitingness for transportation cycling in adults. This is the first study investigating the effect of changing micro-scale environmental factors by using manipulated panoramic photographs. Based upon our quantitative and qualitative data, ‘evenness of the cycle path’ appeared to be the most important perceived environmental factor associated with invitingness to cycle for transportation. Limited research has examined this factor as a potential barrier to cycling. One Canadian study using questionnaire data found that when a route had potholes or uneven paving, the likelihood of cycling declined [45]. Because most European research used the NEWS Questionnaire to assess environmental perceptions [14], where walking/cycling facilities were incorporated together, it was not possible to draw conclusions about the isolating effect of the evenness of the cycle path in these previous studies.

‘General upkeep’ together with ‘separation between cycle path and motorized traffic’ appeared to be the second most important factors to increase the invitingness for transportation cycling. Moreover, both environmental factors interacted with another environmental factor. A well-maintained environment without graffiti on the wall, broken windows, garbage and holes in the road was perceived as more inviting to cycle for transportation compared to a poorly maintained environment. Based on the qualitative data, a large part of the effect of general upkeep is probably explained by the hole in the road surface because it was considered dangerous when a car would avoid it and come closer to the cycle path. The same results were found in a non-European study [46], indicating the importance of good road pavement for cars: the higher the defects scores were of the road surface for motorized traffic, the lower the proportion of adults who cycled to work. ‘General upkeep’ seems to be especially relevant when it causes dangerous situations for cyclists. Furthermore, the positive effect of an even cycle path was stronger in a well-maintained compared to a poorly maintained environment. A combination of these factors could achieve a larger effect on the invitingness of transportation cycling, than to change them separately. Furthermore, the positive effect of having a separation between cycle path and motorized traffic on transportation cycling, was confirmed by previous studies [45,47]. A recent observational study by Sallis and colleagues [29] found that implementing measures to improve cyclists’ safety from cars would increase cycling.

The second interaction effect reported in this study suggest that the positive effect of a separation with traffic could be reduced if there was a separation from the sidewalk as well. A possible reason, provided in the qualitative data, was the frightening feeling for cyclists that would be created when two separations are present on both sides of the cycle path. Another reason may be the choice of using bollards in the photographs to separate cyclists from pedestrians because participants are afraid to cycle against these bollards or see this as a disturbing factor that limited evasive options. This may also explain the non-significant main effect of a ‘separation between cycle path and sidewalk’ on the invitingness for transportation cycling. These results should be approached with caution because the provision of separate cycling facilities was the cornerstone of Dutch, Danish and German policies to make cycling safe and attractive [35]. In these countries, city planners did not use bollards to separate cyclists and pedestrians, but grade separation, pavement coloring or surfacing and mentioned that it is important to present visual and physical, to indicate where cyclists and pedestrians are allowed to travel [48]. It is possible that only pavement coloration, as was present on the pictures too, is enough to make a distinction between cyclists and pedestrians.

In both sorting tasks, the absence of traffic was also an important issue, although many participants are realistic about the necessity of cars and make no claim to get all roads traffic free. The impact of traffic danger has also been mentioned in the literature. Perceived and objective traffic danger have been negatively associated with transportation cycling, both the ‘volume’ (e.g., the street has a lot of motorized traffic) as well as the ‘safety’ aspect (e.g., the risk of collision with automobilists) [45,47]. Nevertheless, a study of Foster and colleagues [18] found no effect of traffic volumes on transportation cycling and appeared to be more strongly related to leisure cycling than to transportation cycling.

The above mentioned significant positive associations of micro-scale modifications like an even cycle path, no obstacles, a separation from motorized traffic and low traffic level with the invitingness for transportation cycling, may have an important effect on safety. Because, safety is shown to be an important determinant regarding whether or not people will cycle [49], those small and easy changes are important to increase cycling, especially in countries where prevalence rates are still low due to lack of safety [35]. These findings may have important policy implications as they suggest that safety measures may be more effective to promote cycling for transportation than measures to improve the aesthetic appeal of a street. However, further research in real-life settings is warranted to find out whether such modifications could change actual cycling behavior.

A wide cycle path, the presence of vegetation and the presence of a speed bump were important for the invitingness, but to a lesser extent compared to the other environmental factors. The qualitative data confirmed that these environmental factors were not considered as a priority for the participants. Previous research shows [48] that the minimum width of cycle tracks should be 78 inches (1.98 m) clear to provide safe passing for cyclists while overtaking another cyclist. Another issue is the various opinions of the participants concerning the presence of trees. The trees were mostly seen as an obstacle for cyclists as well as for pedestrians, that could hinder cyclists while avoiding the trees. Other results might be obtained if the trees would be placed somewhere else. Furthermore, it is difficult to draw conclusions about the aesthetics of vegetation because different types of vegetation were manipulated together in this study. These findings, compared to existing literature, indicate the complexity of the environment. The weak relationship between the presence of a speed bump and the increasing invitingness of transportation cycling could be explained with the help of the qualitative data. Many participants could not make the link between the presence of a speed bump and the advantage for cyclists. In the literature, evidence shows that speed bumps improve safety for cyclists [35]. It might be less important for the increasing invitingness for cyclists, but it still remains an important component regarding the traffic safety.

Finally, no moderating effects of the demographic factors on the relationships between the environmental factors and the invitingness for transportation cycling were found. This finding may be encouraging for planning, because improvements of the micro-environment may have the potential to increase the invitingness of transportation cycling in both genders, the age group (45–65 years) and all educational levels. However, before drawing definite conclusions, these findings need to be replicated in a larger group of middle-aged adults recruited from different geographic areas.

The main strength of the present study was the experimental design, because causal conclusions on the effects of modifications in the environment on the invitingness can be drawn. Furthermore, these findings could be used to develop environmental interventions to determine if these findings will actually change the cycling behavior. A second strength was the collection of both quantitative and qualitative data. The qualitative data could help to figure out the underlying reasons why participants sort the pictures in a certain way. Third, there was the use of the manipulated panoramic photographs that have been validated to on-site responses. This allowed the possibility to ask for more items that were combined together at the same time.

This study also has some limitations that should be acknowledged. First, in this study the relationships with invitingness for transportation cycling was assessed and not with actual cycling behavior. Therefore, the present results can only give suggestions towards developing environmental interventions to determine if these findings will actually change the cycling behavior of adults. Environmental interventions in real life settings are needed to find out whether changing the micro-scale environmental factors, identified in this study, will affect actual cycling behavior. Second, in each sorting task, only five environmental variables could be manipulated. Adding an additional environmental factor would exponentially increase the number of photographs and the burden for the participant. Third, ‘general upkeep’ and ‘vegetation’ are environmental factors, consisting of many subcomponents that were manipulated simultaneously. Consequently, it is impossible to say which of the manipulated elements is crucial for changing the invitingness. Fourth, a limitation of using color photographs is the lack of movement [50]. In real life, people notice different things in the environment depending on their speed of travel. The use of computer-generated virtual walk-through environments could be a suitable solution [51]. Fifth, the study sample was relatively small, which might make the results less generalizable. Therefore, the findings need to be confirmed in larger samples. A last limitation of this study is that only one streetscape was used for this experiment. Consequently, it is not possible to generalize these findings to other streetscapes. In further studies it should be investigated whether the effect of micro-scale environmental factors on the invitingness for transportation cycling depends on macro-scale environmental factors. If micro and macro- environmental factors are interacting, future studies should also include different environmental macro settings, e.g., environments with high versus low land use mix diversity.

## Conclusions

In conclusion, this study has contributed to the research about neighborhood built environment changes to increase the overall PA levels of adults. Our findings indicate that evenness of the cycle path may be a crucial environmental factor when aiming to increase a street’s invitingness for transportation cycling among middle-aged adults. Moreover, the effects might be stronger in a good compared to a poorly maintained environment. In addition, cycling invitingness of the physical environment can be enhanced if there is a ‘separation between cycle path and motorized traffic’ , without the presence of a ‘separation between cycle path and sidewalk’ by means of bollards. Also a low traffic level, a wide cycle path, the presence of a speed bump and vegetation appear to have a positive impact on the invitingness-score for transportation cycling. Furthermore, it is not inviting for transportation cycling to separate cycle path and sidewalk by using bollards. No moderating effects of demographic factors were found. To know whether these results are generalizable to the entire middle-aged adult population, our findings should be confirmed in a larger sample recruited from different geographic areas. On-site research is needed to confirm these current findings.
